# Biodecolorization and Ecotoxicity Abatement of Disperse Dye-Production Wastewater Treatment with *Pycnoporus* Laccase

**DOI:** 10.3390/ijerph19137983

**Published:** 2022-06-29

**Authors:** Bin Wang, Yanjun Chen, Jian Guan, Yiwen Ding, Yide He, Xueying Zhang, Nosir Shukurov, Luiz Fernando Romanholo Ferreira, Jiayang Liu, Mingxin Zhu

**Affiliations:** 1 School of Environmental Science and Engineering, Nanjing Tech University, Nanjing 211816, China; 202061202023@njtech.edu.cn (B.W.); 201921002145@njtech.edu.cn (Y.C.); 201961202013@njtech.edu.cn (J.G.); 202161202017@njtech.edu.cn (Y.D.); heyd@njtech.edu.cn (Y.H.); xueyingzhang@njtech.edu.cn (X.Z.); 2 Institute of Geology and Geophysics, State Committee of the Republic of Uzbekistan on Geology and Mineral Resources 49, Olimlar Street, Tashkent 100041, Uzbekistan; nosirsh@gmail.com; 3 Graduate Program in Process Engineering (PEP), Tiradentes University, Aracaju 49032-490, Brazil; luiz_fernando@itp.org.br; 4 Waste and Effluent Treatment Laboratory, Institute of Technology and Research (ITP), Aracaju 49032-490, Brazil

**Keywords:** laccase, ecotoxicity, decolorization, disperse dye, wheat seed, activated sludge

## Abstract

The biological treatment efficiency of dye wastewater using activated sludge (AS) is largely limited to the chromaticity and ecotoxicity of dyestuff. To alleviate this limitation, eleven industrial-grade disperse dyes were obtained from a fiber-dyeing factory, and for the first time, we studied the decolorization and detoxification effects of using the *Pycnoporus* laccase enzyme. Efficient decolorization was achieved with the following conditions: dye concentration 50 mg/L, 1-hydroxybenzotriazole (HBT) 0.15 mM, temperature 65 °C, pH 4, and laccase 0.33 U/mL. The decolorization rate of disperse dyes, ranging from 51 to 96% in this investigation, was highly dependent on the dye type, concentration, laccase loading, and HBT. The ecotoxicity of dyes was evaluated by studying the germination/growth of wheat seed as well as the respiratory rate of aerobic AS. Laccase treatment mitigated the phytotoxicity of dyes because of the higher wheat germination (e.g., increase of 38% for Black ECT 200%) and growth rate (e.g., increase of 91% for Blue 2BLN 200%). The reduced ecotoxicity of decolorized dye solution towards microorganisms was also confirmed by the finding that the oxygen uptake by aerobic AS was increased relative to that of the untreated samples (e.g., increase of 14 folds for Blue HGL 200%). In addition, the chemical oxygen demand (COD) of decolorized dye solution was slightly lower than that without decolorization during the respiratory test. The experimental results suggest that enzymatic decolorization and detoxification can be potentially used as a pretreatment method for disperse dye wastewater followed by AS treatment.

## 1. Introduction

Synthetic dyes are broadly applied in various fields, e.g., textile, leather, cosmetic, and food and drug industries [1]. There are over 10,000 different dyes on the global scale, 70% of which are azo dyes, amounting to a worldwide production of >1,000,000 tons per year [2]. The available data show that almost all synthetic dyes (especially azo) are eco-toxic and likely to cause cancer and mutations in organisms [3]. According to the applicable properties, they can be classified into acid, basic, direct, disperse, active, and vat dyes [3]. In the textile industry, approximately 10–20% of dyes cannot be fixed onto the textures during the dyeing process, thereby generating a huge amount of dye-containing wastewater [4]. It has been estimated that 1.84 billion tons of textile wastewater were generated in 2015, and it is considered one of the major industrial wastewaters in China [5]. The direct discharge of dye wastewater without treatment may cause severe environmental pollution and ecological issues [6]. A series of treatment methods, such as physical, chemical, and biological treatments, along with their combinations, have been developed and evaluated [7,8]. Recently, increasing interest has been paid to the enzymatic decolorization of synthetic dyes (e.g., laccase) due to numerous advantages—fast reaction, low cost, mild conditions, no generation of undesired by-products such as extra sludge, etc. [9,10].

A number of enzymes have been proven capable of decolorizing synthetic dyes, with laccase being identified as one of the most efficient ones [11]. Laccase (EC 1.10.3.2) is a multi-copper-containing oxidase and is widely distributed in eukaryotic and prokaryotic organisms [12]. Among various sources, fungal laccases have gained a significant amount of interest due to their high redox potential, especially in comparison with bacterial ones [13]. Although decolorization with laccase alone has been well-documented, the presence of mediators, usually small molecules, plays a distinctly subsidiary role in enlarging the substrate spectrum and enhancing the catalytic efficiency [14]. 1-hydroxybenzotriazole (HBT) is one of the most employed and effective mediators [15]. With laccase alone or a laccase mediator system (LMS), the decolorization of numerous dyes has been achieved, but most of them are analytic-grade synthetic dyes [16]. The presence of an acid, base, salt, and mediators in industrial-grade dyes makes it more complex, toxic, and more difficult to be treated or decolorized by laccase or LMS [17]. To the best of our knowledge, the decolorization of industrial-grade dyes (especially disperse dyes) using laccase or LMS has been scarcely reported.

In addition to intensive coloration, toxicity toward the natural ecosystem represents another troublesome problem for dye wastewater. Following decolorization by laccase, detoxification has been found to be simultaneously achieved to some extent [18]. Some prokaryotes and eukaryotes have been employed as model organisms to evaluate the detoxification performance, including bacteria (pure culture, e.g., *Vibrio fischeri*), activated sludge (mixed culture), algae (e.g., *Raphidocelis subcapitata*), plants (e.g., *Triticum aestivum*), animals (mostly protozoan, e.g., *Daphnia magna*), and insect cells [19,20]. In the process of the industrial-scale treatment of dye wastewater, the biological method, i.e., the anaerobic/aerobic activated sludge (AS) method, is widely used as an indispensable part, usually following physical and chemical treatments. These combinations are often designed to remove color and chemical materials from wastewater. The decolorization in such practices can be mostly ascribed to anaerobic AS decomposition and partially to adsorption, which is also time-consuming and inefficient [21]. Furthermore, the performance of chemical oxygen demand (COD) removal by subsequent aerobic AS highly depends on the toxicity and bioavailability of decolorized/degraded dyestuffs from anaerobic AS decomposition. Since laccase has been proven capable of decolorizing synthetic dyes and might be able to replace the anaerobic AS process, there is a great necessity to evaluate the ecotoxicity of decolorized dye wastewater using laccase against aerobic AS. 

Many types of dyes, such as acid, basic, and reactive dyes, have been well-studied by laccase or LMS [19], whereas the performance of the decolorization and detoxification of disperse dyes is unknown. Disperse dyes are generally used for dyeing hydrophobic fibers such as polypropylene and polyester [22]. The present work aimed to evaluate the decolorization efficiency of 11 disperse dyes obtained from a textile factory under varying reaction conditions with a laccase of the genus *Pycnoporus*. Following decolorization under optimal conditions for each dye, the ecotoxicity of the dye solution towards plant seed and AS was assessed, thus providing useful data for practical applications.

## 2. Materials and Methods

### 2.1. Laccase, Chemicals, Wheat Seed, and Activated Sludge

Thermostable laccase enzyme was produced by cultivating robust fungus *Pycnoporus* sp. SYBC-L3 (GenBank access number JX861099) in a bioreactor using submerged mode with detailed information described in our previous report [23]. The culture broth was centrifuged (8000 rpm for 10 min) to produce liquid fraction, which was termed as crude laccase enzyme and used in this study. Mediator 1-hydroxybenzotriazole (HBT) and enzyme-substrate 2,6-dimethoxyphenol (DMP) were procured from Macklin Reagent (Shanghai, China) and Sigma-Aldrich (St. Louis, MO, USA), respectively. The other chemicals were purchased from Sinopharm Group, China. Wheat seeds (labeled as Xinong 529) were obtained from Changfeng Seed Co., Ltd. (Shanxi, China). Eleven synthetic dyes of industrial grade were gifted from a fiber dyeing company (Huzhou, China) with the available information described in Table 1. The maximum absorbance wavelength of each dye solution in water was obtained by a UV-Vis spectrophotometer. Activated sludge (AS) was collected from a secondary clarifier in a local municipal wastewater treatment plant in Nanjing, China. 

### 2.2. Measurement of Laccase Activity 

In light of the high cost of purified laccase in practical applications, crude laccase as prepared in the above section was used in this study. The original activity of the crude laccase was determined as 33 U/mL. Laccase activity was spectrophotometrically determined with DMP (ε = 49.5 mM^−1^ cm^−1^) as substrate. The increase in absorption at 470 nm was recorded in a reaction system, as previously described [23]. One unit of laccase activity corresponded to the amount of enzyme that oxidized 1 μmol of DMP per min. The reaction system of 3 mL consisted of 2.4 mL citric-phosphate buffer (20 mM, pH = 3), 0.5 mL DMP solution (10 mM), and 0.1 mL enzyme solution. 

### 2.3. Decolorization of Disperse Dyes

Compared to other types of synthetic dyes, disperse dyes are nonionic and show poor solubility in water due to hydrophobic functional groups [22]. In this study, the eleven industrial-grade disperse dyes were found to have good solubility in water at the experimental concentrations (25–100 mg/L). UV-Vis spectrophotometer was therefore employed to obtain the characteristic absorption peak by wavelength scanning of each dye solution at 50 mg/L, based on which calibration curve of each dye was obtained for further dye concentration determination in various treatments. The decolorization reaction was carried out in 250 mL flask that contained dye solution (25–100 mg/L), buffer (pH 3–8), laccase (0.165–3.3 U/mL), HBT (0.205–0.5 mM), and at temperature ranging from 25–80 °C. Upon completion of the reaction, the residual dye concentration in the supernatant was analyzed with a UV-Vis spectrophotometer. Decolorization rate (color removal) was calculated as follows: R (%) = [(Ai − At)/Ai] × 100, where Ai and At are the initial and final absorption values at peak wavelength for each dye, respectively [24]. 

The time course of decolorization within 3 h was obtained by recording the absorbance value of the reaction system every 30 min at each optimal reaction condition. The effect of pH values ranging from 3.0 to 8.0 on decolorization was studied by fixing other variables at optimal reaction conditions. Mediator HBT was added to the reaction system to study its effect on decolorization rate with other variables fixed at optimal reaction conditions. The effect of temperature on decolorization was carried out in the same way as described above. 

### 2.4. Seed Germination and Growth Experiment

The pH values of both pretreated and untreated dye solutions were adjusted to 7.0 using 1 mol/L NaOH or HCl before the phytotoxicity experiment [25]. Decolorized dye solution was obtained under its optimal condition, as confirmed above. A total of 10 carefully selected intact wheat seeds, soaked in 3% H_2_O_2_ for 30 min in advance to disinfect the potential undesired microbes, were evenly placed onto the double-layered filter paper in a 9 cm glass Petri dish with sterilized water added for adequate seed imbibing. Three replicates were performed for water or each dye solution of a different treatment. A total of 5 mL of variously treated dye solution, sterilized using autoclave at 120 °C for 20 min to avoid microbial contamination, was added to each plate every 24 h. The plate was put into a thermostat incubator (25 ± 0.5 °C) for 7 d, where the first three days were under dark conditions and the latter four days under 12 h light and 12 h dark conditions. After the cultivation, the germination rate and shoot and root length of the sprout were recorded. Based on the data of shoot and root length of wheat sprout in water, the percent growth inhibition of wheat sprout in various dye solutions was calculated. 

### 2.5. Oxygen Uptake by Aerobic Activated Sludge (AS)

Freshly collected AS from the municipal wastewater treatment plant (as described in Section 2.1) was aerated in the laboratory for 2 h to allow the depletion of residual nutrients in the AS. Subsequently, the AS was characterized as follows: sludge volume (SV 30) 21%, mixed liquid suspended solids (MLSS) 5900 mg/L, and sludge volume index (SVI) 35.6 mL/g. These data indicated the good settling performance of AS. To conduct the oxygen uptake experiment, three different treatment of each dye (concentration of 100 mg/L) was designed: un-decolorization with heat deactivated laccase, decolorization with only laccase, and decolorization with laccase + HBT, respectively. The decolorization treatment was completed under the optimal condition for each dye as identified in this study. The dye solution (250 mL) of different treatments was then mixed with AS at a volume ratio of 1:1, roughly giving rise to the final AS concentration (MLSS) of 2950 mg/L, which was close to the real AS concentration in industries. The mixed solution of total 500 mL was then adjusted to pH 7 and put in a bottle, which was connected to the PF–8000 aerobic/anaerobic respirometer system [26]. The oxygen uptake in each bottle was automatically recorded online over the time course of 12 h. In addition, the chemical oxygen demand of dye solution before and after the respiratory experiment was determined using the potassium dichromate method.

### 2.6. Statistical Analyses 

Each experiment, including the decolorization and ecotoxicity test, was carried out in triplicate. The mean value plus standard deviation (SD) was calculated and presented in figures and tables. 

## 3. Results

### 3.1. Visible Absorption Spectrum of Disperse Dyes

**Figure 1:**
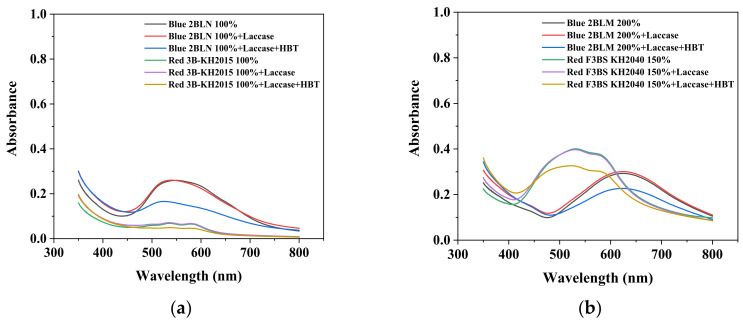

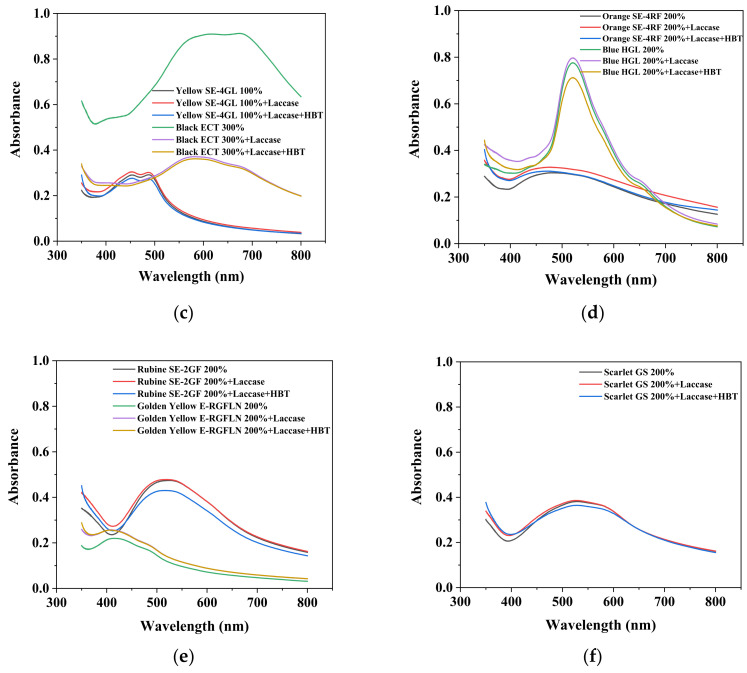
UV-Vis spectra of disperse dye solution (50 mg/L) before and after laccase (0.165 U/mL) or laccase (0.165 U/mL) + HBT (0.15 mmol/L) treatment. pH of each solution was natural and temperature was 25 °C. (**a**): Blue 2BLN 100% and Red 3B-KH2015 100%; (**b**): Blue 2BLM 200% and Red F3BS KH2040 150%; (**c**): Yellow SE-4GL 100% and Black ECT 300%; (**d**): Orange SE-4RF 200% and Blue HGL 200%; (**e**): Rubine SE-2GF 200% and Golden Yellow E-RGFLN 200%; (**f**): Scarlet GS 200%.

Eleven industrial-grade dyestuffs, all belonging to disperse dyes, were obtained from a textile dyeing factory (Table 1). According to the information on the packages, four dyes were further confirmed in detail concerning the chemical structures and dye classes: Disperse Scarlet GS 200% (azo), Disperse Blue HGL 200% (azo), Disperse Golden Yellow E-RGFLN 200% (azo), and Disperse Blue 2BLN 100% (anthraquinone). The detailed information on the remaining seven dyes was not available in the present study. Before the decolorization and ecotoxicity experiments, the visible absorption spectra of the dye solution in water were characterized, and the results are shown in Figure 1. Very broad absorption bands were found for most dye solutions. Some did not even show obvious absorption peaks, e.g., an extremely flat peak for Red 3B-KH2015 100% and Orange SE-4RF 200%, suggesting poor absorption ability. Double peaks were observed for the dye Yellow SE-4GL 100%, while only one peak showed for the other dyes. As seen in Figure 1, the characteristic absorption peak of each dye was identified (Table 1) and employed to determine the dye concentration using the corresponding calibration curve. 

It can be seen from Figure 1 that the absorbance values of most dyes decreased to varying degrees at the individual maximal absorption wavelength, with the exception of Golden Yellow E-RGFLN 200% and Orange SE-4RF 200%, which showed a slight increase in the absorbance. This indicated that the majority of disperse dyes could be successfully decolorized by laccase alone or laccase + HBT. The most significant decolorization was found for Black ECT 300% using laccase with or without the addition of mediator HBT, followed by Blue 2BLN 100% and 200%, Red F3BS KH2040 150%, and Red 3B-KH2015 100%. 

### 3.2. Effect of Different Parameters on Decolorization Rate

**Figure 2:**
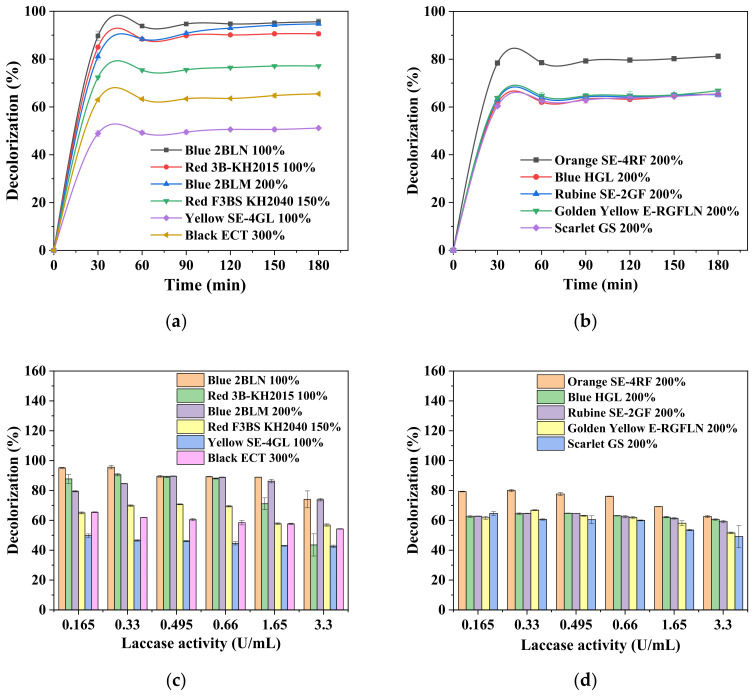
Time course of decolorization rate of 11 disperse dyes (**a**,**b**) under optimal reaction conditions listed in Table 1. Effect of laccase loading (0.165–3.3 U/mL) on decolorization of 11 disperse dyes with other parameters at optimal conditions (**c**,**d**).

The time course of the decolorization rate of various dye solutions under the optimized reaction conditions (Table 1) is displayed in Figure 2a,b. Despite the variation in the chemical structure and composition of dyes, they were all decolorized effectively with laccase or laccase + HBT treatment, ranging from 50% to 95% within 3 h. It was also noted that the equilibrium of decolorization was reached in only 30 min, and afterward, no further decolorization took place. A relatively higher rate of decolorization (>85%) occurred on Blue 2BLN 100%, Blue 2BLM 200%, and Red 3B-KH2015 100%, while the lowest was observed for Yellow SE-4GL 100% (i.e., only 50%). It can be observed that there were still eight dye solutions with a low decolorization rate of <80%.

The effect of laccase dosage on decolorization rate is shown in Figure 2c,d. Previous reports found that increased laccase activity could enhance decolorization to varying degrees, which was, however, not consistent in this study. In contrast, no significant enhancement in the decolorization rate could be seen with the laccase dosage tested, starting from 0.165 to 3.3 U/mL. Higher laccase application even slightly reduced the decolorization rate for some dye solutions, e.g., Orange SE-4RF 200% and Blue 2BLN 100%. This might be ascribed to the introduction of colored substances in the crude laccase that led to the absorption increase at the characteristic wavelength. A practical application might consider a lower laccase dosage for cost-reduction benefits.

**Figure 3:**
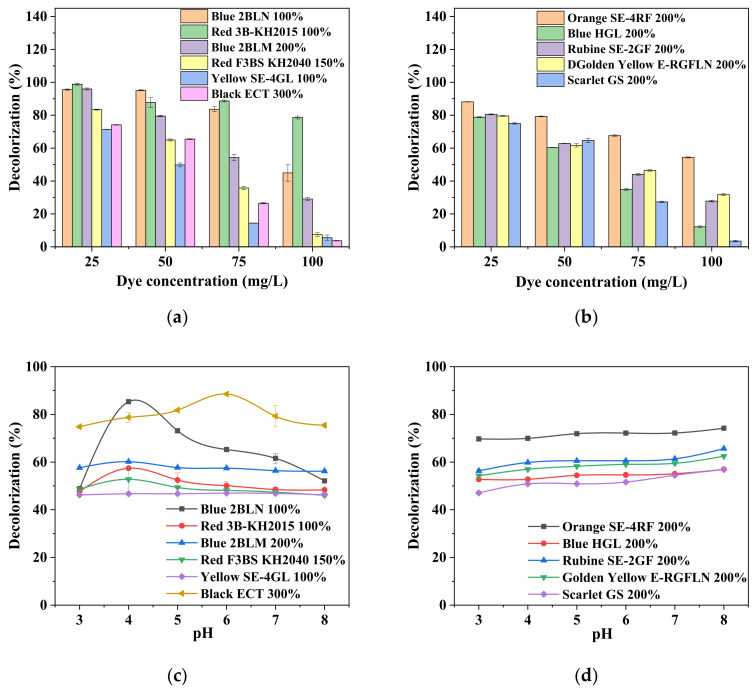
Effect of initial dye concentration (**a**,**b**) and pH value (**c**,**d**) on decolorization rate of 11 disperse dyes with other parameters at optimal conditions, as listed in Table 1.

The initial dye concentration was a major parameter affecting the decolorization rate, as demonstrated in Figure 3a,b. A sharp descending trend was observed for decolorization when a high concentration was applied. Specifically, over 70% decolorization (mostly around 80%) was achieved for dye solutions at 25 mg/L, while 80% decolorization (mostly below 60%) remained for Red 3B-KH 100% when the dye concentration was set to 100 mg/L. In comparison with the dye concentration, the pH value seemed to influence the decolorization rate in a very weak manner, except for dye Blue 2BLN 100% and Black ECT 300%, whose optimal pH was found to be 4 and 6, respectively (Figure 3c,d). 

**Figure 4:**
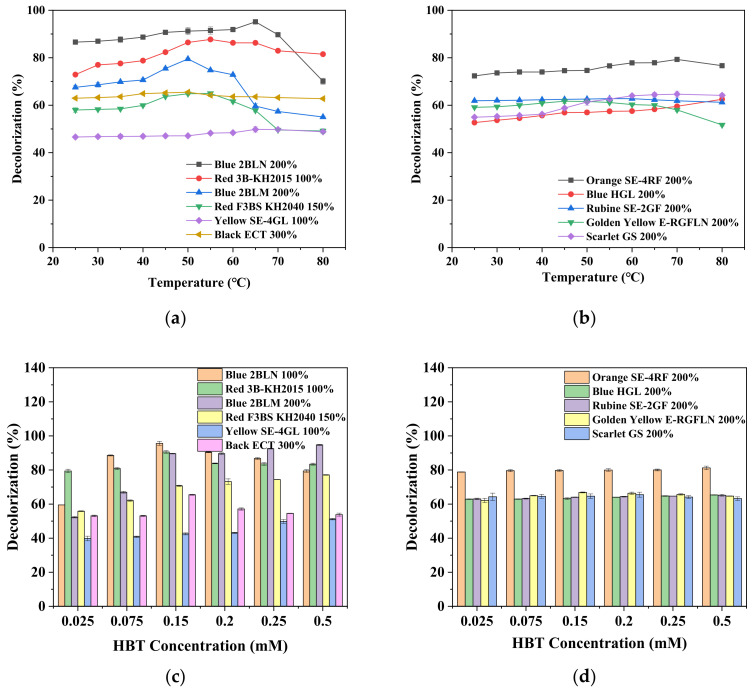
Effect of temperature (**a**,**b**) and HBT dosage (0.025~0.5 mM) (**c**,**d**) on decolorization rate of 11 synthetic dyes with other parameters at optimal conditions, as listed in Table 1.

Similar to the pH value, the temperature of the dye solution influenced the decolorization rate insignificantly (Figure 4a,b). With the exception of Blue 2BLN 100% and Blue 2BLM 200%, whose optimal temperature was 65 °C and 50 °C, respectively, the others kept a constant decolorization rate within the temperature range of 40–80 °C. HBT is one of the most commonly used mediators, and its effect on the decolorization rate is presented in Figure 4c,d. Generally, a positive effect was found for HBT (especially at a lower dosage) on the decolorization rate of 11 disperse dyes, in particular with 6 dye solutions, namely, Blue 2BLN 100%, Red 3B-KH2015 100%, Blue 2BLM 200%, Red F3BS KH2040 150%, Yellow SE-4GL 100%, and Black ECT 300%. The highest concentration of HBT, however, did not result in an increase in the decolorization efficiency; rather, a decreased or no effect was observed, especially in cases with a concentration higher than 0.2 mM, e.g., Blue 2BLN 100%, Red 3B-KH2015 100%, and Black ECT 300%. No significant improvement in the decolorization efficiency was observed for the other five dyes: Orange SE-4RF 200%, Blue HGL 200%, Rubine SE-2GF 200%, Golden Yellow E-RGFLN 200%, and Scarlet GS 200%.

### 3.3. Wheat Seed Germination and Growth in Different Dye Solution

**Figure 5:**
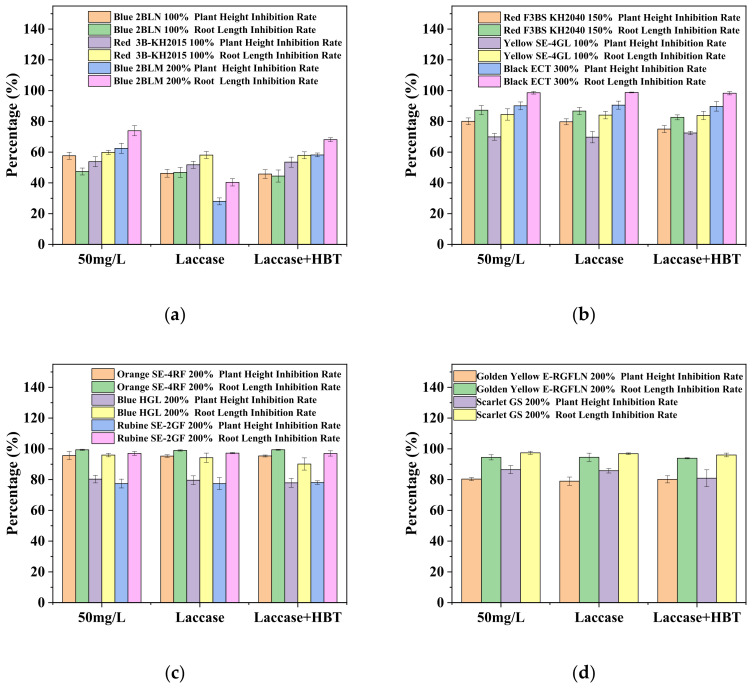
Inhibition rate of shoot and root elongation of wheat sprout under different treatments: deactivated laccase, laccase, and laccase + HBT. (**a**): Blue 2BLN 100%, Red 3B-KH2015 100%, and Blue 2BLM 200%; (**b**): Red F3BS KH2040 150%, Yellow SE-4GL 100%, and Black ECT 300%; (**c**): Orange SE-4RF 200%, Blue HGL 200%, and Rubine SE-2GF 200%; (**d**): Golden Yellow E-RGFLN 200% and Scarlet GS 200%.3.4. Oxygen Uptake by Aerobic AS in Different Dye Solutions.

To reveal the potential environmental effects, phytotoxicity toward wheat seed germination and growth in different dye solutions was determined, and the results are shown in Table 2 and Figure 5. Over 93% wheat seed germination was achieved in water, while in the un-decolorized dye solution, the germination rates were reduced to 17–90%, with most around 50%. The most severe inhibition was found for dye Black ECT 300% and Orange SE-4RF 200%, whose germination was as low as 26.7% and 16.7%, respectively. This demonstrated that the original disperse dye solution had an extremely hazardous impact on seed germination.

Compared with the original dye solution, laccase or laccase + HBT treatment upgraded the germination rate to varying degrees, which was still lower, however, than those in the water treatment. Additionally, in Table 2, elongated roots and shoots can be observed on wheat sprouts cultivated in the laccase or laccase + HBT-treated dye solution compared with those in untreated ones, indicating that the toxicity of decolorized dye solution applied to young plants was mitigated. As portrayed in Figure 5, the percent inhibition towards the shoot and root length was found to be slightly mitigated when exposing the wheat seed to laccase or laccase + HBT treated dye solution. The strongest mitigation inhibition was for Blue 2BLM 200% with laccase treated alone, which was reduced by >50% relative to the untreated sample.

**Figure 6:**
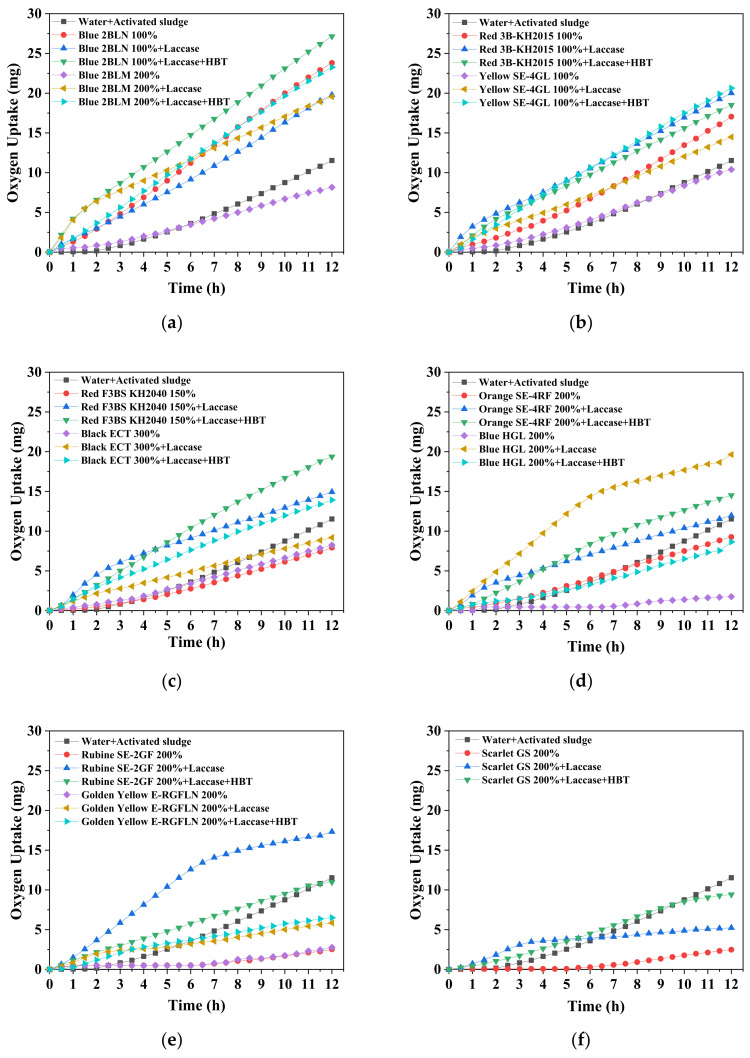
Oxygen uptake by aerobic AS exposed to 11 synthetic dye solutions under different treatments: deactivated laccase, laccase, and laccase + HBT. (**a**): Blue 2BLN 100% and Blue 2BLM 200%; (**b**): Red 3B-KH2015 100% and Yellow SE-4GL 100%; (**c**): Red F3BS KH2040 150% and Black ECT 300%; (**d**): Orange SE-4RF 200% and Blue HGL 200%; (**e**): Rubine SE-2GF 200% and Golden Yellow E-RGFLN 200%; (**f**): Scarlet GS 200%.

Under the best decolorization conditions as determined above (Table 1), the initial dye solution was subjected to the following treatments: dye + deactivated laccase, dye + laccase, and dye + laccase + HBT. These treatments were then mixed with AS for the respiratory experiment. Within 12 h of continuous aeration, oxygen uptake was accordingly attained (Figure 6). With respect to the dye solution without decolorization (dye + deactivated laccase), an obvious inhibition of AS respiration was observed in eight dyes compared to those in water, namely, Blue 2BLN 100%, Red F3BS kH2040 150%, Black ECT 300%, Orange SE-4RF 200%, Blue HGL 200%, Rubine SE-2GF 200%, Golden Yellow E-RGFLN 200%, and Scarlet GS 200%, respectively, among which the last four dyes showed the strongest inhibition because of the lowest oxygen uptake. A relatively insignificant inhibition was found with the other three dyes: Blue 2BLN 100%, Red SB-KH2015 100%, and Yellow SE-4GL 100%.

Following decolorization with laccase or laccase + HBT, aerobic AS was exposed to different dye solutions or water to study the change in oxygen uptake by AS. A drastic alleviation in inhibition of AS was achieved for all the dyes. The increased oxygen uptake at the end of 12 h cultivation was in the range of 20% (Red 3B-KH2015 100% with laccase treatment alone) to 20 folds (Blue HGL 200% with laccase treatment alone) relative to the value of AS exposed to water (i.e., 12 mg oxygen). This demonstrated that laccase-catalyzed decolorization could remarkably alleviate the ecotoxicity of dyes toward AS. The discrepancy, however, was also observed among different dyes depending on dye type.

Furthermore, the COD value of the dye solution before and after the AS respiratory experiment was determined (Table 3). Most dye solutions of laccase-catalyzed decolorization were found to have lowered COD compared with their un-decolorized counterparts, except for three dye solutions, i.e., Blue 2BLM 200%, Yellow SE-4GL 100%, and Golden Yellow E-RGFLN 200%, which showed increased COD. The phenomenon of COD’s increase might be ascribed to the lysis of bacterial cells when exposed to highly toxic dye molecules, resulting in the release of intracellular constituents. The highest COD removal occurred with Red 3B-KH2015 100% (83.3%), Blue 2BLM 200% (38.9%), Yellow SE-4GL 100% 9 (34.4%), and Golden Yellow E-RGFLN 200% (5.72%) of laccase-catalyzed treatments, respectively.

## 4. Discussion

Azo dyes comprise the largest proportion of the global synthetic dyes, followed by anthraquinones and others [27], which was reflected well in the field investigation in this study. Compared to the reported dyes [28], most disperse dye solutions showed very broad absorption bands rather than narrow ones, except for Blue HGL 200%, suggesting that there could be a complex composition of industrial-grade dyestuff, usually supplemented to enhance the color strength and dye fixation [21]. Even though there is a significant number of articles reporting the decolorization of various dyes (mostly acid, basic, direct, and active) by laccase [9,29,30], the description of real dye wastewater or industrial-grade dyes is very scarce, especially for disperse dyes. Relative to simulated dye wastewaters, real textile wastewater or industrial-grade dyes show higher recalcitrance towards decolorization due to the complexity in composition that is usually unfriendly to enzymes. For example, 15% decolorization was achieved following 2 h treatment with *Lentimus* laccase and 4.5% with *Trametes* laccase [25]. Even if the incubation time was elongated to 24 h, only 43% decolorization of these dye effluents was attained [25].

Reaction parameters, e.g., time, pH, temperature, enzyme loading, mediator, and dye concentration, have been reported to influence decolorization significantly. For all disperse dyes, the highest decolorization was achieved around 30 min, which was similar to other reports of simulated dye wastewater [31]. A low decolorization rate has been observed for some dyes in this study, which was lower than the previous results of simulated dye solution [32], indicating that the other components, e.g., the presence of an acid, base, salt, and mediators, in industrial-grade dyes could negatively interfere with laccase-catalyzed decolorization. Some of these substances are reported to have an inhibitory effect on laccase activity and stability [12,33]. An enzyme loading of around 0.17 U/mL was proved to be effective for an optimized decolorization of dispersed dyes herein, which was in the range of 0.2–2 U/mL of enzyme for efficient decolorization [31,32]. Decreased decolorization has been confirmed along with the increased dye concentration, which is in good agreement with previous results [34]. The effect of pH on the decolorization of disperse dyes is consistent with the previous results that the best decolorization would occur around pH 5, while a pH above 6 or below 3 is not favorable to laccase-catalyzed decolorization [32]. These results are in favor of practical applications because there will be no need to further adjust the pH of wastewater with acid or alkaline to cater to laccase that is normally catalytically active in acidic conditions [35]. Efficient decolorization normally occurs at temperatures ranging from 40 to 60 °C [36], beyond which decolorization drops rapidly. Considering the effluent from the dyeing house usually has a high temperature of up to 80 °C, and most fungal laccase has an optimal temperature at 50–70 °C, it is possible to employ thermostable laccase to decolorize effluent instantly collected from the dyeing house. Generally, the decolorization of disperse dyes by laccase enzyme exhibited similar behavior to other types of synthetic dyes.

According to the field investigation, it is common that the direct discharge of untreated dye wastewater to the natural water body or its direct use as water for crop irrigation still exists in some regions of China. This suggests that synthetic dyes have a significantly detrimental effect on seed germination and growth [25]. In analytical-grade dyes (e.g., AB80 and AR37), their phytotoxicity toward rice germination and growth was almost completely relieved after laccase-catalyzed decolorization, i.e., equivalent to that in water [25], whereas in this study, such a change was relatively little, strongly indicating that components other than the dye molecules in the industrial-grade dyes could exert extra toxicity towards seeds and plants. Similar results—a slight alleviation in phytotoxicity towards *Lepidium sativum*—have been described for the fungus-induced decolorization of ABBB and AB129 in a recent study [16]. Interestingly, a decolorized dye solution of ABBB or AB129 seemed to cause a lower germination index (GI) than the initial dye solutions [16]. These results clearly demonstrate that huge discrepancies could be obtained in terms of the ecotoxicity evaluation when applying different testing organisms.

Based on the amount of the accumulated oxygen uptake, an inhibitory (less oxygen uptake) or stimulatory (more oxygen uptake) effect of the chemicals on AS respiration could be reflected [26]. Generally, the decolorization of disperse dyes by laccase led to less ecotoxicity and thus stimulated AS growth and metabolism, as reflected in the increased oxygen demand. Nonetheless, the different chemical structures of dyes could have varied the toxicity toward AS. In practice, dyes with higher toxicity toward AS can be collected and pretreated separately by laccase or LMS to lower their toxicity, thus enhancing the efficiency of the following biological units, i.e., the aerobic AS process. After the AS respiratory experiment, the COD of the decolorized solution showed lower values compared with those of the un-decolorized solution. The possible reason for this is that the degraded dye products were presumably readily absorbed and metabolized in bacterial cells due to the reduced toxicity. Another possible reason for this result was that a small part of organics, such as dye molecules in this study, might be transformed into H_2_O and CO_2_ by laccase with the presence of molecular oxygen [37].

Although the detoxification of a dye solution using laccase treatment has been confirmed, it is unknown if laccase treatment favors the subsequent AS-involved biological unit in the practical dye wastewater treatment process. A similar concept has been attempted, however. Manai et al. introduced a fungal ligninolytic enzyme directly into a CSTR tank containing real textile effluent fed with AS recycled from a secondary clarifier and elevated COD removal up to 95% from the control value of 75% [38]. Additionally, an improvement in color removal and strong resistance to shock loadings of pollutants, as well as the augmented sludge volume index (SVI) and microbial activity, have been observed simultaneously [38]. The results in this study and other reports strongly imply that there is potential for the laccase pretreatment of dye wastewater to achieve both effective decolorization and detoxification, thus improving the general treatment efficiency of dye wastewater.

## 5. Conclusions

The current study, for the first time, investigated the efficiency of the decolorization and detoxification of an industrial-grade disperse dye in an aqueous solution with an enzyme laccase alone or with a laccase-mediator system (LMS). Compared to other types of synthetic dyes, disperse dyes were more resistant to enzymatic decolorization. Generally, a decolorization rate of 51% to 96% was achieved in this study, which largely depended on various parameters, including the dye type, dye concentration, laccase loading, and HBT. Along with the decolorization, a notable reduction in the ecotoxicity of decolorized dyes towards plant seed and aerobic microbes was observed. During the respiratory test of aerobic AS, lowered COD values were found in most laccase-decolorized dye solutions compared with the un-decolorization sample, suggesting that laccase-catalyzed decolorization can improve the subsequent efficiency of the AS process and therefore potentially serve as an effective pretreatment approach for dye wastewater treatment.

## Figures and Tables

**Table 1:** Description of 11 disperse dyes from a textile factory. ‘/’ means currently unknown in this study.

Dye Name	λ_max_ (nm)	MW (g/mol)	CAS	Chemical Formula	Structure	Classification	Optimal Condition for Decolorization
Blue 2BLN 100%	547	304.69	12217-79-7	C_14_H_9_ClN_2_O_4_	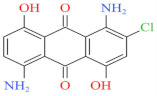	Anthraquinone	pH 4, 65 °C, laccase 0.33 U/mL, HBT 0.15 mM, decolorization 95.6%
Red 3B-KH2015 100%	583	/	/	/	/	/	pH 4, 55 °C, laccase 0.33 U/mL, HBT 0.15 mM, decolorization 90.6%
Blue 2BLM 200%	629	/	/	/	/	/	pH 4, 50 °C, laccase 0.50 U/mL, HBT 0.50 mM, decolorization 94.74%
Red F3BS KH2040 150%	526	/	/	/	/	/	pH 4, 55 °C, laccase 0.50 U/mL, HBT 0.50 mM, decolorization 77.1%
Yellow SE-4GL 100%	486	/	/	/	/	/	pH 6, 65 °C, laccase 0.17 U/mL, HBT 0.50 mM, decolorization 51.2%
Black ECT 300%	580	/	/	/	/	/	pH 6, 50 °C, laccase 0.17 U/mL, HBT 0.15 mM, decolorization 65.5%
Orange SE-4RF 200%	488	/	/	/	/	/	pH 8, 70 °C, laccase 0.33 U/mL, HBT 0.50 mM, decolorization 81.2%
Blue HGL 200%	520	639.41	12239-34-8	C_24_H_27_BrN_6_O_10_	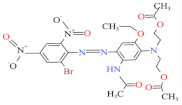	Azo	pH 8, 80 °C, laccase 0.50 U/mL, HBT 0.50 mM, decolorization 65.4%
Rubine SE-2GF 200%	522	/	/	/	/	/	pH 8, 55 °C, laccase 0.50 U/mL, HBT 0.50 mM, decolorization 65.2%
Golden Yellow E-RGFLN 200%	417	302.33	6250-23-3	C_18_H_14_N_4_O	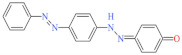	Azo	pH 8, 45 °C, laccase 0.33 U/mL, HBT 0.15 mM, decolorization 66.8%
Scarlet GS 200%	527	404.32	78564-87-1	C_18_H_15_Cl_2_N_5_S	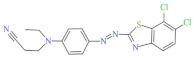	Azo	pH 8, 70 °C, laccase 0.17 U/mL, HBT 0.20 mM, decolorization 65.5%

**Table 2:** Germination rate and root and shoot length of wheat seed exposed to dye solution with or without laccase decolorization. The initial dye concentration was 50 mg/L, and the decolorization of each dye was achieved under its respective optimized conditions.

Dye		Germination Rate (%)				Root Length (cm)				Shoot Length (cm)		
	Water	Un-Treated	Laccase + HBT	Laccase	Water	Un-Treated	Laccase + HBT	Laccase	Water	Un-Treated	Laccase + HBT	Laccase
Blue 2BLN 100%	93.3 ± 9.4	76.7 ± 4.7	80 ± 16.3	76.7 ± 4.7	3.24 ± 1.1	1.45 ± 0.2	1.51 ± 0.3	1.47 ± 0.1	2.23 ± 0.7	0.8 ± 0.1	0.9 ± 0.3	0.95 ± 0.2
Red 3B-KH2015 100%	96.7 ± 4.7	93.3 ± 4.7	93.3 ± 4.7	96.7 ± 4.7	4.33 ± 0.3	1.72 ± 0.3	1.82 ± 0.1	1.82 ± 0.2	3.52 ± 0.7	1.56 ± 0.6	1.57 ± 0.1	1.62 ± 0.2
Blue 2BLM 200%	93.3 ± 4.7	83.8 ± 4.7	86.7 ± 4.7	96.7 ± 4.7	4.16 ± 0.4	1.13 ± 0.2	1.38 ± 0.4	2.6 ± 0.4	3.34 ± 0.3	1.29 ± 0.2	1.45 ± 0.2	2.47 ± 0.9
Red F3BS KH2040 150%	100.0 ± 0	60.0 ± 8.1	66.7 ± 17.0	66.3 ± 12.5	4.53 ± 1.1	0.56 ± 0.1	0.72 ± 0.1	0.58 ± 0.1	4.12 ± 1.5	0.71 ± 0.1	0.87 ± 0.1	0.73 ± 0.1
Yellow SE-4GL 100%	96.7 ± 4.7	90.0 ± 8.2	99.3 ± 4.7	86.7 ± 4.7	4.54 ± 1.2	0.66 ± 0.03	0.69 ± 0.1	0.68 ± 0.03	4.36 ± 1.6	0.95 ± 0.3	0.99 ± 0.2	1.12 ± 0.2
Black ECT 300%	90.0 ± 8.2	26.7 ± 9.4	36.7 ± 9.4	33.3 ± 12.5	7.94 ± 2.5	0.09 ± 0.03	0.11 ± 0.04	0.1 ± 0.05	8.78 ± 1.0	0.83 ± 0.5	0.88 ± 0.3	0.84 ± 0.3
Orange SE-4RF 200%	93.3 ± 4.7	16.7 ± 4.7	23.3 ± 4.7	20.0 ± 0.7	7.25 ± 2.4	0.04 ± 0.02	0.04 ± 0.01	0.07 ± 0.01	8.48 ± 1.7	0.39 ± 0.2	0.4 ± 0.02	0.40 ± 0.03
Blue HGL 200%	96.7 ± 4.7	60.0 ± 0.5	66.7 ± 9.3	63.3 ± 4.7	6.21 ± 1.6	0.23 ± 0.03	0.55 ± 0.1	0.40 ± 0.3	6.25 ± 1.2	0.95 ± 0.2	1.06 ± 0.1	1.01 ± 0.2
Rubine SE-2GF 200%	90.0 ± 8.2	50.0 ± 14.1	60.0 ± 14.1	53.3 ± 18.9	6.15 ± 1.0	0.16 ± 0.04	0.20 ± 0.1	0.17 ± 0.05	5.10 ± 1.3	1.08 ± 0.4	1.1 ± 0.5	1.09 ± 0.3
Golden Yellow E-RGFLN 200%	100.0 ± 0	70.0 ± 14.1	70.0 ± 14.1	66.7 ± 20.6	7.26 ± 0.1	0.42 ± 0.14	0.46 ± 0.04	0.43 ± 0.2	8.01 ± 0.2	1.60 ± 0.1	1.62 ± 0.2	1.73 ± 0.6
Scarlet GS 200%	100.0 ± 0	55.3 ± 12.5	63.3 ± 18.9	56.7 ± 17.1	7.52 ± 0.7	0.19 ± 0.07	0.29 ± 0.1	0.23 ± 0.02	8.14 ± 0.4	1.09 ± 0.2	1.53 ± 0.4	1.16 ± 0.2

**Table 3:** Variation in COD (mg/L) of dye solution before and after AS oxygen uptake experiment. The initial dye concentration was 50 mg/L, which was treated with laccase or laccase + HBT. Treatments a, b, and c represent un-decolorized dye solution with heat-deactivated laccase followed by AS, decolorized dye solution with only laccase followed by AS, and decolorized dye solution with laccase + HBT followed by AS, respectively.

Dye	Treatment ^a^		Treatment ^b^		Treatment ^c^	
	COD_cr_ (mg/L) Before	COD_cr_ (mg/L) After	COD_cr_ (mg/L) Before	COD_cr_ (mg/L) After	COD_cr_ (mg/L) Before	COD_cr_ (mg/L) After
Blue 2BLN 100%	112.33 ± 2.05	97.67 ± 2.05	377 ± 2.94	197.67 ± 2.03	217 ± 2.16	147.67 ± 2.05
Red 3B-KH2015 100%	416 ± 2.94	67.12 ± 2.16	77.67 ± 2.06	47.67 ± 2.04	207 ± 2.45	112.33 ± 1.70
Blue 2BLM 200%	56 ± 2.74	77.67 ± 2.05	187.67 ± 2.01	77.67 ± 2.04	289.67 ± 1.25	177 ± 2.16
Red F3BS KH2040 150%	107.67 ± 2.05	58 ± 2.16	127.66 ± 2.04	97.67 ± 1.70	292.33 ± 1.70	270 ± 1.63
Yellow SE-4GL 100%	127.33 ± 2.09	207.67 ± 1.70	177.68 ± 2.09	61 ± 0.82	270 ± 1.63	177 ± 2.16
Black ECT 300%	147.67 ± 2.07	57.33 ± 2.05	126.67 ± 2.13	102 ± 2.16	260.67 ± 1.70	138 ± 2.14
Orange SE-4RF 200%	77.67 ± 2.03	67 ± 2.16	87.76 ± 2.32	77.67 ± 2.05	249 ± 2.94	188 ± 1.63
Blue HGL 200%	177.67 ± 2.06	70 ± 1.63	236.77 ± 2.49	108.33 ± 1.70	297.67 ± 2.05	287.33 ± 2.05
Rubine SE-2GF 200%	187 ± 2.16	56.33 ± 2.62	467.32 ± 2.03	138.33 ± 1.72	279.67 ± 2.87	262.33 ± 1.70
Golden Yellow E-RGFLN 200%	57.67 ± 2.05	477.67 ± 2.05	137.43 ± 2.07	127 ± 2.45	116.67 ± 2.87	110 ± 1.63
Scarlet GS 200%	17.67 ± 2.05	17.33 ± 1.25	87 ± 2.16	67 ± 2.16	149.33 ± 2.49	90.33 ± 1.25

## Data Availability

The data presented in this study are available in this article.

## References

[1] Tkaczyk A., Mitrowska K., Posyniak A. (2020). Synthetic organic dyes as contaminants of the aquatic environment and their implications for ecosystems: A review. Sci. Total Environ..

[2] Das A., Mishra S. (2017). Removal of textile dye reactive green-19 using bacterial consortium: Process optimization using response surface methodology and kinetics study. J. Environ. Chem. Eng..

[3] Croce R., Cinà F., Lombardo A., Crispeyn G., Cappelli C.I., Vian M., Maiorana S., Benfenati E., Baderna D. (2017). Aquatic toxicity of several textile dye formulations: Acute and chronic assays with *Daphnia magna* and *Raphidocelis subcapitata*. Ecotoxicol. Environ. Saf..

[4] Cui M.-H., Cui D., Gao L., Wang A.-J., Cheng H.-Y. (2016). Azo dye decolorization in an up-flow bioelectrochemical reactor with domestic wastewater as a cost-effective yet highly efficient electron donor source. Water Res..

[5] Liang J., Ning X.-A., Sun J., Song J., Lu J., Cai H., Hong Y. (2018). Toxicity evaluation of textile dyeing effluent and its possible relationship with chemical oxygen demand. Ecotoxicol. Environ. Saf..

[6] Coria-Oriundo L.L., Battaglini F., Wirth S.A. (2021). Efficient decolorization of recalcitrant dyes at neutral/alkaline pH by a new bacterial laccase-mediator system. Ecotoxicol. Environ. Saf..

[7] Criado S.P., Gonçalves M.J., Tavares L.B.B., Bertoli S.L. (2020). Optimization of electrocoagulation process for disperse and reactive dyes using the response surface method with reuse application. J. Clean. Prod..

[8] Kishor R., Purchase D., Saratale G.D., Saratale R.G., Ferreira L.F.R., Bilal M., Chandra R., Bharagava R.N. (2021). Ecotoxicological and health concerns of persistent coloring pollutants of textile industry wastewater and treatment approaches for environmental safety. J. Environ. Chem. Eng..

[9] Iark D., Buzzo A.J.D.R., Garcia J.A.A., Côrrea V.G., Helm C.V., Corrêa R.C.G., Peralta R.A., Moreira R.D.F.P.M., Bracht A., Peralta R.M. (2019). Enzymatic degradation and detoxification of azo dye congo red by a new laccase from *Oudemansiella canarii*. Bioresour. Technol..

[10] Ali H. (2010). Biodegradation of synthetic dyes—A review. Water Air Soil Pollut..

[11] Choi K.-Y. (2020). Discoloration of indigo dyes by eco-friendly biocatalysts. Dye. Pigment..

[12] Baldrian P. (2006). Fungal laccases—Occurrence and properties. FEMS Microbiol. Rev..

[13] Vilar D.D.S., Bilal M., Bharagava R.N., Kumar A., Nadda A.K., Salazar-Banda G.R., Eguiluz K.I.B., Ferreira L.F.R. (2021). Lignin-modifying enzymes: A green and environmental responsive technology for organic compound degradation. J. Chem. Technol. Biotechnol..

[14] Claus H., Faber G., König H. (2002). Redox-mediated decolorization of synthetic dyes by fungal laccases. Appl. Microbiol. Biotechnol..

[15] Husain M., Husain Q. (2007). Applications of redox mediators in the treatment of organic pollutants by using oxidoreductive enzymes: A review. Crit. Rev. Environ. Sci. Technol..

[16] Rybczyńska-Tkaczyk K., Korniłłowicz-Kowalska T., Szychowski K., Gmiński J. (2020). Biotransformation and toxicity effect of monoanthraquinone dyes during *Bjerkandera adusta* CCBAS 930 cultures. Ecotoxicol. Environ. Saf..

[17] Yagub M.T., Sen T.K., Afroze S., Ang H.M. (2014). Dye and its removal from aqueous solution by adsorption: A review. Adv. Colloid Interface Sci..

[18] Becker D., Della Giustina S.V., Rodriguez-Mozaz S., Schoevaart R., Barceló D., De Cazes M., Belleville M.-P., Sanchez-Marcano J., De Gunzburg J., Couillerot O. (2016). Removal of antibiotics in wastewater by enzymatic treatment with fungal laccase—Degradation of compounds does not always eliminate toxicity. Bioresour. Technol..

[19] Gao Y., Wang M., Shah K., Kalra S.S., Rome L.H., Mahendra S. (2022). Decolorization and detoxification of synthetic dye compounds by laccase immobilized in vault nanoparticles. Bioresour. Technol..

[20] Novotný C., Dias N., Kapanen A., Malachová K., Vandrovcova M., Itävaara M., Lima N. (2006). Comparative use of bacterial, algal and protozoan tests to study toxicity of azo- and anthraquinone dyes. Chemosphere.

[21] Routoula E., Patwardhan S.V. (2020). Degradation of anthraquinone dyes from effluents: A review focusing on enzymatic dye degradation with industrial potential. Environ. Sci. Technol..

[22] Penthala R., Park S.H., Oh H., Lee I.Y., Ko E.H., Son Y.-A. (2022). An ecofriendly dyeing of nylon and cotton fabrics in supercritical CO_2_ with novel tricyanopyrrolidone reactive disperse dye. J. CO_2_ Util..

[23] Liu J., Yu Z., Liao X., Liu J., Mao F., Huang Q. (2016). Scalable production, fast purification, and spray drying of native *Pycnoporus* laccase and circular dichroism characterization. J. Clean. Prod..

[24] Abadulla E., Tzanov T., Costa S., Robra K.-H., Cavaco-Paulo A., Gübitz G.M. (2000). Decolorization and detoxification of textile dyes with a laccase from *Trametes hirsuta*. Appl. Environ. Microbiol..

[25] Hsu C.-A., Wen T.-N., Su Y.-C., Jiang Z.-B., Chen C.-W., Shyur L.-F. (2012). Biological degradation of anthroquinone and azo dyes by a novel laccase from *Lentinus* sp.. Environ. Sci. Technol..

[26] Liu X., Huang F., Yu Y., Jiang Y., Zhao K., He Y., Xu Y., Zhang Y. (2019). Determination and toxicity evaluation of the generated byproducts from sulfamethazine degradation during catalytic oxidation process. Chemosphere.

[27] Tan L., He M., Song L., Fu X., Shi S. (2016). Aerobic decolorization, degradation and detoxification of azo dyes by a newly isolated salt-tolerant yeast *Scheffersomyces spartinae* TLHS-SF1. Bioresour. Technol..

[28] Singh R.L., Singh P.K., Singh R.P. (2015). Enzymatic decolorization and degradation of azo dyes—A review. Int. Biodeterior. Biodegrad..

[29] Debnath R., Mistry P., Roy P., Roy B., Saha T. (2021). Partial purification and characterization of a thermophilic and alkali-stable laccase of *Phoma herbarum* isolate KU4 with dye-decolorization efficiency. Prep. Biochem. Biotechnol..

[30] Espina G., Cáceres-Moreno P., Mejías-Navarrete G., Ji M., Sun J., Blamey J.M. (2020). A novel and highly active recombinant spore-coat bacterial laccase, able to rapidly biodecolorize azo, triarylmethane and anthraquinonic dyestuffs. Int. J. Biol. Macromol..

[31] Zimbardi A.L.R.L., Camargo P.F., Carli S., Neto S.A., Meleiro L.P., Rosa J.C., De Andrade A.R., Jorge J.A., Furriel R.P.M. (2016). A high redox potential laccase from *Pycnoporus sanguineus* RP15: Potential application for dye decolorization. Int. J. Mol. Sci..

[32] Mechichi T., Mhiri N., Sayadi S. (2006). Remazol brilliant blue R decolourization by the laccase from *Trametes trogii*. Chemosphere.

[33] Sen S.K., Raut S., Bandyopadhyay P., Raut S. (2016). Fungal decolouration and degradation of azo dyes: A review. Fungal Biol. Rev..

[34] Sayahi E., Ladhari N., Mechichi T., Sakli F. (2015). Azo dyes decolourization by the laccase from *Trametes trogii*. J. Text. Inst..

[35] Morsi R., Bilal M., Iqbal H.M., Ashraf S.S. (2020). Laccases and peroxidases: The smart, greener and futuristic biocatalytic tools to mitigate recalcitrant emerging pollutants. Sci. Total Environ..

[36] Zeng X., Cai Y., Liao X., Zeng X., Li W., Zhang D. (2011). Decolorization of synthetic dyes by crude laccase from a newly isolated *Trametes trogii* strain cultivated on solid agro-industrial residue. J. Hazard. Mater..

[37] Nakamura Y., Mtui G. (2003). Biodegradation of endocrine-disrupting phenolic compounds using laccase followed by activated sludge treatment. Biotechnol. Bioprocess Eng..

[38] Manai I., Miladi B., El Mselmi A., Hamdi M., Bouallagui H. (2016). Improvement of activated sludge resistance to shock loading by fungal enzyme addition during textile wastewater treatment. Environ. Technol..

